# Pangolin-Associated Viruses: Diversity, Evolutionary Adaptation, and Zoonotic Spillover Risks

**DOI:** 10.3390/ani16193077

**Published:** 2026-09-30

**Authors:** Cheng Yu, Yaoming Wu, Xiaoya Huang, Fanghui Mao, Xuelin Wang, Rongquan Zheng, Xiaoyong Chen

**Affiliations:** 1 Xingzhi College, Zhejiang Normal University, Jinhua 321100, China; y1419251326@163.com (C.Y.); yaomingwu2026@163.com (Y.W.); 19700939659@163.com (X.H.); 13757267175@163.com (F.M.); 13857229794@163.com (X.W.); 2 College of Life Sciences, Zhejiang Normal University, Jinhua 321004, China

**Keywords:** pangolin, viral diversity, viral evolution, host adaptation, zoonotic spillover risk

## Abstract

Pangolin-associated viruses have received considerable attention because of their diversity and potential zoonotic relevance, yet the evidence remains fragmented. This review evaluates the diversity, evolutionary adaptation, and zoonotic spillover risks of pangolin-associated viruses. Diverse RNA and DNA viruses, such as coronaviruses, paramyxoviruses, astroviruses, parvoviruses, and papillomaviruses, have been detected in pangolins, showing substantial taxonomic and genomic variation. Studies of selected pangolin-associated viral groups have documented sequence variation and identified signals of recombination or reassortment. Some pangolin coronaviruses can use human receptors and replicate in human-derived cells, organoids, or transgenic animal models. Several viruses known to infect humans have also been detected in pangolin samples, although conclusive evidence of natural spillover from pangolins to humans is lacking. Most studies examine Sunda and Chinese pangolins from illegal trade or rescue centers, relying largely on nucleic acid detection and sequence analysis. Habitat disruption, wildlife trafficking, long-distance transport and captive-rescue operations may increase cross-host–pathogen exposure. Future work should prioritize surveillance of wild pangolin populations of known provenance and integrate virus isolation, tissue localization, serology, and functional assays to support pangolin conservation and reliable zoonotic-risk evaluation.

## Simple Summary

Pangolins are highly endangered wild mammals threatened by illegal wildlife trade. Multiple viruses have been detected in pangolins, some of which raise public-health concerns due to their potential to spread between animals and humans. Most virological studies have examined rescued or confiscated pangolins involved in illegal trade, and data from wild pangolin populations remain scarce. This review summarizes the diverse viruses detected in pangolins, describes their evolutionary changes and host-adaptation features, and evaluates their potential for cross-species spillover to humans. Several pangolin-derived coronaviruses and other viruses can replicate in human-derived cells or laboratory animal models, yet natural transmission of viruses from pangolins to humans has not been demonstrated. Habitat loss, long-distance trafficking, animal stress during transport, and rescue and captive-care settings may increase pathogen exposure risks for both animals and people. Better monitoring of well-characterized wild populations, stricter control of illegal wildlife trade, and improved biosecurity in rescue centers will benefit pangolin conservation and help reduce zoonotic disease risks.

## 1. Introduction

Pangolins are specialized scale-covered mammals belonging to the family *Manidae* within the order *Pholidota*. According to the taxonomy adopted by the International Union for Conservation of Nature (IUCN), the eight extant pangolin species are classified into three genera—*Manis*, *Phataginus*, and *Smutsia*—and are distributed across Asia and sub-Saharan Africa [1,2]. Long-term anthropogenic pressures such as illegal hunting and habitat loss have caused sustained declines in pangolin population sizes and distribution ranges [3]. Currently, all eight pangolin species are listed as threatened on the IUCN Red List, and their conservation status remains critical [1]. In China, the national protection status of all pangolin species in the genus *Manis* was upgraded from Class II to Class I in 2020. The updated National List of Key Protected Wildlife in 2021 further specified that the Chinese pangolin (*Manis pentadactyla*), Sunda pangolin (*Manis javanica*), and Indian pangolin (*Manis crassicaudata*) are listed as Class I nationally protected wildlife [4].

In recent years, pangolin conservation efforts have gradually expanded beyond trade regulation and legal protection to include field population surveys and long-term monitoring, habitat protection, the rescue and release of confiscated individuals, and ex situ breeding [5,6,7]. In this process, infectious diseases have been recognized as an important factor affecting individual survival and ex situ conservation management [8,9]. Previous virological investigations of rescued, diseased, or deceased pangolins have identified multiple types of RNA and DNA viruses, including paramyxoviruses, coronaviruses, orthopestivirus-like viruses, and reovirus-related viruses [10,11,12]. More recent studies have shown that smuggled pangolins can be co-infected with multiple viruses and other pathogens, accompanied by varying degrees of tissue damage [9]. Since coronaviruses related to severe acute respiratory syndrome coronavirus 2 (SARS-CoV-2) were detected in and isolated from Sunda pangolins in 2020, the potential public health relevance of pangolin-associated viruses has attracted considerable attention [13,14,15]. Subsequently, with the application of techniques such as metagenomics, metatranscriptomics, and virus isolation, research on pangolin-associated viruses has rapidly expanded beyond the detection of a few viruses to encompass multiple areas, including virome diversity, host distribution, viral evolutionary relationships, and cross-species infection potential [12,16].

Previous syntheses have examined the biological characteristics of SARS-CoV-2 and COVID-19 [17], as well as the diversity, distribution, and infection potential of pangolin-associated microbes [18]. Building on these studies, this review updates and synthesizes research on pangolin-associated RNA and DNA viruses, focusing on viral diversity and host associations, evolutionary dynamics and host adaptation, and evidence for cross-species infection. This synthesis further considers how ecological and anthropogenic factors, including wildlife trade, may influence viral exposure and transmission, integrating these factors with virological evidence to inform pangolin conservation, viral surveillance, and zoonotic risk assessment.

## 2. Diversity of Pangolin-Associated Viruses

**Figure 1:**
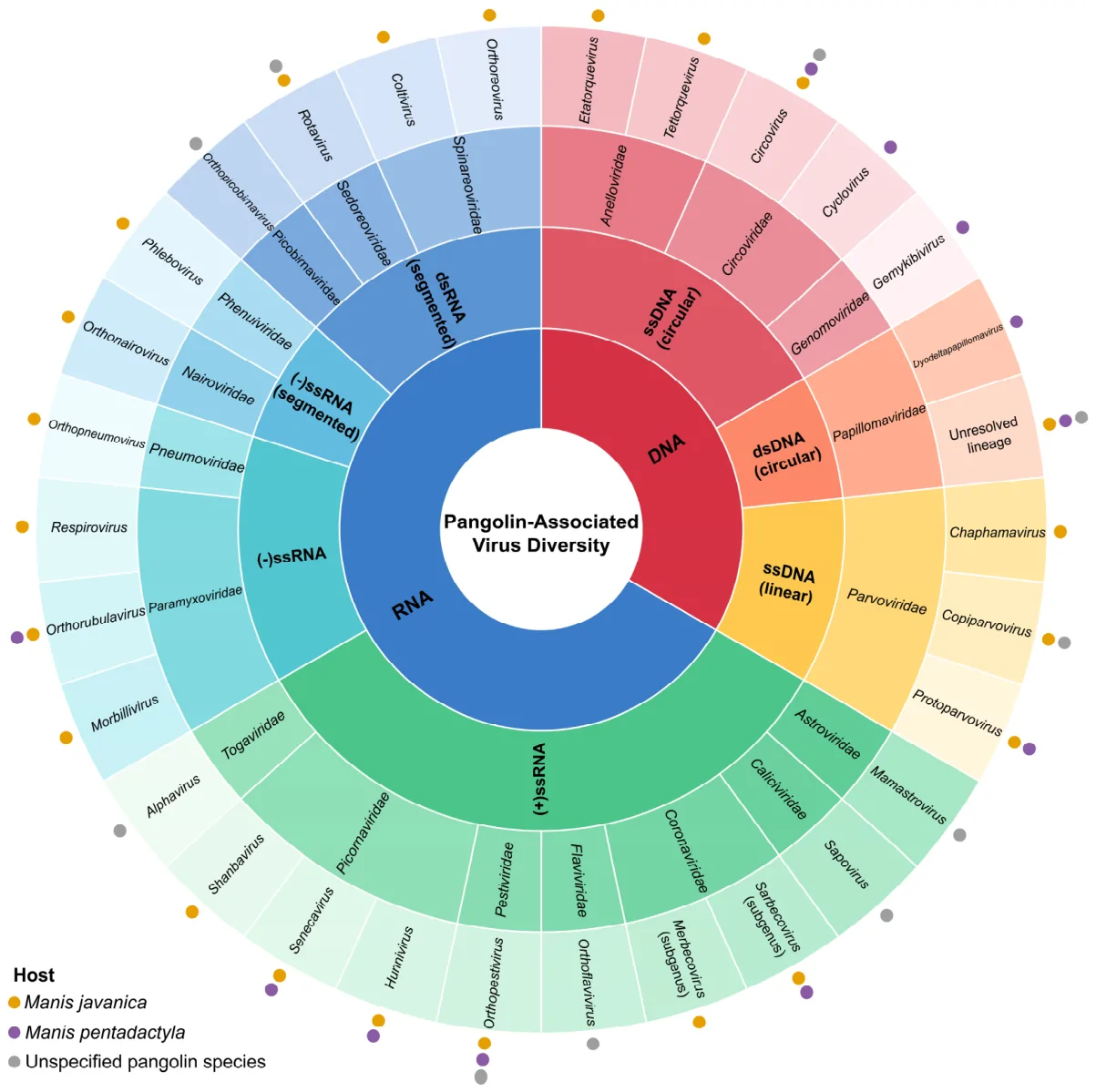
Genomic and taxonomic diversity of pangolin-associated viruses. From the center outward, the rings represent nucleic acid type, genome type, viral family, and genus, subgenus, or unresolved lineage. Terminal taxa are equally weighted to emphasize diversity rather than viral abundance or detection frequency. Colors distinguish genome types and their nested taxa. Outer dots indicate reported associations with *Manis javanica* (orange), *Manis pentadactyla* (purple), or unspecified pangolin species (gray). ss, single-stranded; ds, double-stranded; (+), positive-sense; (−), negative-sense.

Based on literature searches of relevant databases and supplementary manual searches, this review compiles published virological studies on pangolins, with the search covering publications up to August 2026. The detailed literature-search strategy and study-selection process are provided in Table S1. Viral records were included primarily from peer-reviewed studies that provided clear information on pangolin sample origins and at least one type of evidence supporting host association, including nucleic acid detection or genome sequences, serological or histopathological evidence, virus isolation, or experimental infection. For viruses identified through metagenomic, metatranscriptomic, or host genomic data mining, only records with unambiguous pangolin origins and traceable sequence information were included. This review identified 30 taxonomic units at the genus, subgenus, or unassigned-lineage level, representing 19 virus families. The host associations and supporting evidence for these 30 taxonomic units are summarized in Table S2. Because most available evidence comes from trafficked, confiscated, or rescued pangolins, the findings summarized below primarily reflect the viruses detected in these individuals and cannot be directly generalized to viral diversity in free-ranging pangolin populations. The identified viruses represented five major genome types: positive-sense single-stranded RNA, negative-sense single-stranded RNA, double-stranded RNA, single-stranded DNA, and double-stranded DNA (Figure 1). Among these, coronaviruses have become one of the most intensively studied groups in pangolin-associated virus research, owing to their relatively well-established evidence base, including complete genome sequences, phylogenetic analyses, virus isolation, and partial functional data. Pangolin coronaviruses also include members of the subgenera *Sarbecovirus* and *Merbecovirus*, which contain important human pathogens [15,19].

### 2.1. Coronaviruses

Coronaviruses are enveloped RNA viruses with broad host ranges, and some can cross species barriers and cause respiratory or gastrointestinal disease [17]. To date, pangolin-associated coronaviruses with complete genomes and systematic characterization belong mainly to the genus *Betacoronavirus*, spanning two subgenera, *Sarbecovirus* and *Merbecovirus* [12,14]. The former includes pangolin-associated coronaviruses closely related to SARS-CoV-2 and constitutes the main focus of current pangolin coronavirus research; pangolin-associated members of the latter are represented by recently identified HKU4-related coronaviruses and belong to the same subgenus as Middle East respiratory syndrome coronavirus (MERS-CoV), further expanding the known lineage spectrum of pangolin coronaviruses [20].

Early evidence of pangolin-associated sarbecoviruses came from viromic investigations of deceased and rescued individuals. In 2019, viral metagenomic sequencing conducted on tissues from a deceased Sunda pangolin detected coronavirus-related sequences [10]. Following the onset of the coronavirus disease 2019 pandemic, reanalysis of the publicly available metagenomic data from lung tissues further identified viral sequences with high homology to SARS-CoV-2 [21]. In 2020, multiple studies obtained relatively complete genomes of pangolin SARS-CoV-2-related coronaviruses and successfully isolated viruses, showing that these viruses could be classified primarily into two genetic lineages, Guangdong-related and Guangxi-related, and that they are closely related phylogenetically to SARS-CoV-2 and various bat-derived SARS-related coronaviruses [14,15,22]. With the expansion of research, these viruses have been detected in tissue, swab, and excretion samples from trafficked, rescued, and captive pangolins [23,24]. Their reported host range has also expanded from Sunda pangolins to Chinese pangolins [25]. It should be noted that screening results vary across regions and source populations. Viral nucleic acids have been detected in trafficked, rescued, or captive pangolins in China and Vietnam [23,24], and serological responses have also been reported in pangolins originating from wildlife checkpoints in Thailand [26]. By contrast, large-scale screening of confiscated and rescued Sunda pangolins from Malaysia did not detect coronaviruses [27]. These observations suggest that the available data are significantly influenced by host species, geographic region, and sampling context, and are thus insufficient to reflect the true prevalence of sarbecoviruses in natural pangolin populations. In addition to sarbecoviruses, a metatranscriptomic analysis conducted on smuggled Sunda pangolins in 2022 also identified, for the first time, a virus in the subgenus *Merbecovirus* that was closely related to bat HKU4-CoV, which was subsequently named pangolin-CoV-HKU4-P251T [12]. Subsequently, MjHKU4r-CoV, a closely related virus identified in another group of Sunda pangolins, was characterized using polymerase chain reaction (PCR), serology, complete-genome sequencing, and virus isolation [19]. Building on this work, further studies successfully isolated a live virus from the original samples in which P251T was initially detected. The isolated virus shared 99.1% nucleotide identity with MjHKU4r-CoV-1, thereby linking the findings from two independent batches of pangolin samples and supporting the infection of Sunda pangolins with closely related HKU4-related merbecoviruses [28].

### 2.2. Other Pangolin-Associated Viruses

#### 2.2.1. Other RNA Viruses

With advances in metatranscriptomic and viromic technologies, knowledge of RNA viruses in pangolins has gradually expanded from early sporadic detections to systematic descriptions of multiple viral groups and their host-association characteristics. Multiple studies have shown that different viral groups exhibit diverse host association patterns. Viruses within the genera *Orthopestivirus* and *Hunnivirus* typically form relatively independent pangolin-associated lineages [12,16,29]. In contrast, some respiratory RNA viruses detected in pangolin samples are closely related to known human viruses, mainly including human respiratory syncytial virus (HRSV), human parainfluenza virus 3 (HPIV3), and human parainfluenza virus 2 (HPIV2) [9,30,31,32]. Additionally, some viruses are phylogenetically more closely related to other mammalian or arthropod-associated viruses, including viruses assigned to or related to the genera *Respirovirus* and *Mamastrovirus*, as well as parainfluenza virus 5 (PIV5) [33,34,35], mosquito-borne viruses such as Japanese encephalitis virus (JEV), chikungunya virus (CHIKV), and Getah virus (GETV) [36], as well as phleboviruses and orthonairoviruses that are closely related to known tick-associated viruses [12]. In addition, viruses related to the genus *Orthopicobirnavirus* and rotavirus A have close relatives from multiple host species and therefore do not exhibit a clear, single host-association pattern [12,34]. Notably, multiple types of RNA viruses have also been detected in healthy pangolins, including orthopestiviruses, hunniviruses, mamastroviruses, orthopicobirnavirus-related viruses, rotavirus A, JEV, CHIKV, and GETV, indicating that the diversity of pangolin RNA viruses is not confined to diseased or deceased individuals [34,36,37].

Research on a few pangolin RNA viruses has progressed beyond sequence discovery. A pangolin-derived *Respirovirus* has been isolated and evaluated in mouse infection and contact-transmission experiments [33]. Studies of *Orthopestivirus* have combined isolation with preliminary host-range testing in mammalian species and cell lines [16], while studies of pangolin-associated HPIV2 have included targeted PCR, phylogenetic analysis, and viral abundance comparisons [9]. At present, however, except for the few viruses for which virus isolation or additional evidence of infection has been obtained, most reported pangolin RNA viruses remain characterized primarily by sequence discovery, genome assembly, and phylogenetic analysis, whereas pathobiological evidence—such as virus isolation, tissue tropism, experimental infection, and transmissibility—remains limited [38].

#### 2.2.2. DNA Viruses

Research on DNA viruses in pangolins is even more limited than research on RNA viruses, with existing records primarily involving the families *Parvoviridae* and *Circoviridae*, as well as several papillomavirus lineages in the family *Papillomaviridae* [12,34,37,39]. Among these, canine parvovirus type 2 (CPV-2) has been studied more extensively, with histopathological and virological evidence supporting an association between infection and disease. This virus has been detected in both Sunda and Chinese pangolins [12,40]. Some infected individuals have been shown to develop severe necrotizing enteritis characterized by necrosis of intestinal crypts, with supporting evidence obtained from histopathology, immunohistochemistry, and in situ hybridization [41]. In addition, further studies have successfully isolated CPV-2a and CPV-2c [42]. Phylogenetic analyses showed that pangolin-derived CPV-2 is closely related to strains from canids and to strains circulating in surrounding regions, suggesting possible cross-species transmission between canids and pangolins [40,42]. In contrast, research on *Circoviridae* and *Papillomaviridae* has so far been based primarily on virome sequencing and host genomic data mining. Although multiple complete viral genomes have been obtained and their genetic diversity and phylogenetic relationships have been preliminarily characterized, further evidence from virus isolation, tissue tropism, and experimental pathogenicity studies remains lacking [34,37,39], while research on other DNA viruses has remained largely at the stage of metagenomic sequence discovery and preliminary genomic characterization [12,43]. Overall, current studies have preliminarily revealed the diversity of pangolin DNA viruses through sequence discovery and genomic characterization. However, with the exception of a few viruses such as CPV-2, the natural host associations, tissue tropism, pathogenicity, and transmissibility of most of these viruses remain largely unverified.

### 2.3. Overall Disease Evidence and Knowledge Gaps

The available studies indicate that the depth of evidence for pangolin-associated viruses varies considerably. A substantial proportion of viral records are still based primarily on nucleic acid and sequence evidence obtained through metagenomic, metatranscriptomic, or targeted PCR approaches [18]. These studies have effectively expanded our understanding of viral diversity in pangolins, but the detection of viral nucleic acids alone is insufficient to demonstrate that a virus is infectious, capable of sustained replication in pangolin hosts, or causally associated with the observed disease [44].

A fundamental limitation of current pangolin virological research is the predominance of samples from smuggled, confiscated, or rescued individuals, together with the concentration of studies on Sunda and Chinese pangolins [18]. This sampling bias limits inference about the natural pangolin virome, particularly its composition and the prevalence of individual viruses in free-ranging populations. Smuggling, long-distance transport, and intensive confinement alter the original patterns of host contact and pathogen exposure in pangolins, meaning that infection records from confiscated individuals may reflect influences from both natural ecology and trade processes and are therefore more difficult to interpret in terms of virus–host associations and disease links [45]. Even when a virus is abundant in individuals with pneumonia, hemorrhage, or multiorgan damage, the observed disease cannot be attributed to that virus on this basis alone. Instead, a comprehensive assessment integrating viral load, tissue localization, pathological changes, the presence of other pathogens, and the individual’s overall health status is required [9,46]. Furthermore, because the true geographic origins and timing of infection in smuggled individuals are generally difficult to reconstruct, viruses detected after confiscation often cannot be definitively attributed to pre-existing infections acquired in natural habitats, infections contracted during transport, or subsequent exposure in rescue settings [12]. This issue is particularly prominent for viruses related to human pathogens: their detection does not exclude the possibility of human-to-pangolin transmission during trade, captivity, or rescue, making it difficult to determine whether pangolins serve as natural or maintenance hosts or merely as susceptible hosts that experience sporadic infections under specific exposure conditions [30]. Therefore, there is an urgent need to expand long-term surveillance of well-defined wild populations and other pangolin species, integrating healthy controls, multi-tissue sampling, serology, virus isolation, and experimental infection approaches, in order to distinguish between viral detection, true infection, and causal relationships with disease, and to establish a more reliable baseline for pangolin viral ecology and disease.

## 3. Evolutionary Dynamics and Host Adaptation

Pangolin-associated viruses exhibit diverse host-association and phylogenetic patterns. Some viruses form relatively independent pangolin-associated clades in phylogenetic trees, including certain lineages within the genera *Orthopestivirus* and *Hunnivirus* and several pangolin-associated papillomavirus lineages, suggesting some degree of host-associated divergence [12,39]. Others are closely related to viruses originating from bats, humans, canids, or other mammals, as exemplified by pangolin-associated sarbecoviruses, HKU4-related merbecoviruses, HRSV, and CPV-2. Such close relationships suggest that their evolution may have involved cross-species transmission, common ancestry, and a complex history of host contacts [12,14]. Overall, the viral diversity currently observed in pangolins is more likely the result of multiple processes acting in concert, including long-term genetic variation, viral transmission among different hosts, and genetic exchange [47]. Within this phylogenetic framework, clarifying how these genetic differences arose and which changes may influence the capacity of viruses to infect and persist in different hosts is essential for understanding the evolution and host adaptation of pangolin-associated viruses [48].

### 3.1. Evolutionary Mechanisms Generating Viral Diversity

Viral genetic diversity can be shaped by multiple processes, including sequence mutations that accumulate continuously during replication, as well as recombination and reassortment in segmented viruses [49,50,51]. Although these processes do not follow a fixed evolutionary direction, they can nevertheless give rise to genetic changes that may affect antigenicity, host adaptation, or transmissibility, thereby providing a potential basis for the emergence of higher-risk phenotypes [52,53].

Several forms of sequence variation have been observed in pangolin-associated RNA viruses. In PIV5-GD18, comparative genomic analysis identified four nucleotide substitutions resulting in four amino acid changes distributed across the hemagglutinin–neuraminidase, L, and M proteins [35]. In pangolin-associated hunniviruses, all seven complete genomes examined lacked the L-protein gene, and three deletions totaling 82–85 amino acids were identified in the 2B protein [12]. Pangolin-associated orthopestiviruses also exhibit marked interstrain variation in NS5 sequences and form a distinct phylogenetic clade sister to pestiviruses from artiodactyls [16]. Pangolin SARS-CoV-2-related coronaviruses exhibit divergence between the Guangdong- and Guangxi-related lineages and intra-host variation, while low genome-wide ratios of nonsynonymous to synonymous substitution rates indicate strong purifying selection [25]. Functional studies have shown that a lysine at spike residue 519 in some Guangdong pangolin coronaviruses can alter spike conformation and reduce susceptibility to antibody neutralization [54]. These findings illustrate the functional consequences of sequence variation but do not establish that the substitution arose within pangolins or enhanced viral infectivity or host adaptation. Beyond sequence variation, genomic analyses have revealed signals consistent with recombination or reassortment in selected pangolin-associated viral groups. A putative recombination event has been identified in the Guangxi-related pangolin coronavirus sublineage [25], while comparative analyses of pangolin, bat, and human sarbecoviruses support a broader history of recombination within this subgenus [14,55]. Evidence of reassortment in pangolin orthoreovirus comes from discordant phylogenetic relationships among genome segments: L1, L2, and S4 cluster with a bat-derived strain, M3 and S2 with civet- and pig-derived strains, and M2 and S3 with a tree-shrew-derived strain [12]. Analyses including pangolin-derived sequences have also identified three candidate recombination events in *Parvoviridae* and *Paramyxoviridae*. In one example, a dog-derived *Morbillivirus canis* sequence clustered with another dog-derived sequence outside the candidate recombinant region, but with a pangolin-derived sequence within that region, providing phylogenetic support for recombination [37].

Overall, the studies reviewed here document sequence variation and provide evidence for recombination or reassortment in selected pangolin-associated viral groups. The available evidence varies in both type and depth across viral groups. Furthermore, given that the sample sources, timing of infection, and host contact history in some studies are difficult to trace, the host background and transmission pathways of the relevant evolutionary events remain difficult to accurately reconstruct [12]. Determining which of these genetic changes can alter viral host range, infectivity, or transmission phenotypes will require further functional evidence from studies of cell infection, receptor utilization, viral replication, and animal infection [56].

### 3.2. Molecular and Phenotypic Features Associated with Host Adaptation

Functional studies of selected pangolin-associated viruses have characterized receptor compatibility, host protease-dependent entry, and replication or transmission in experimental models. Among these studies, evidence concerning receptors and cell-entry mechanisms is concentrated primarily on coronaviruses, whereas the potential host ranges of other viruses are reflected mostly indirectly by experimental infection phenotypes [19,57,58].

The cross-species receptor compatibility of coronaviruses is one of the best-characterized features to date. The receptor-binding domains of pangolin coronaviruses from the Guangxi- and Guangdong-related lineages can bind to both pangolin and human angiotensin-converting enzyme 2 (ACE2), and exhibit receptor-binding patterns similar to those of SARS-CoV-2 [57,59]. Among the representative viruses studied, MpCoV-GX, from the Guangxi lineage, can utilize ACE2 from humans, pangolins, civets, pigs, mice, and bats to mediate cell entry and can infect cell lines derived from multiple mammalian species. Evidence of infection has also been obtained in three mouse models [60]. PgCoV GD, from the Guangdong lineage, similarly exhibits broad ACE2 receptor usage and can replicate in primary human nasal and airway epithelial cells and alveolar organoids. It can also infect mice and hamsters and undergo relatively inefficient airborne transmission among hamsters [58]. Pangolin HKU4-related coronavirus MjHKU4r-CoV-1 can utilize dipeptidyl peptidase 4 (DPP4) from a wide range of mammals—including pigs, goats, cats, rabbits, macaques, sheep, camels, marmosets, and cattle—to mediate infection. It has also been shown to replicate in human airway and intestinal organoids, and to establish infection and induce pathological changes in the lungs of transgenic mice expressing human DPP4. Compared with the known bat HKU4-CoV, the spike protein of MjHKU4r-CoV-1 possesses an RQQR furin cleavage site, which promotes spike pre-cleavage and reduces the dependence of viral entry on exogenous trypsin, thereby enhancing its utilization of host endogenous proteases [19]. In addition to coronaviruses, some pangolin-associated viruses have also demonstrated cross-host replication phenotypes in heterologous animal models. For instance, a virus in the genus *Respirovirus* isolated from Sunda pangolins can replicate in the respiratory tract and multiple tissues of mice, cause marked pulmonary lesions, and be transmitted through contact between mice, indicating that this isolate can complete its replication cycle and undergo individual-to-individual transmission in a heterologous mammalian host [33].

Taken together, these studies indicate that some pangolin-associated viruses possess molecular prerequisites for overcoming certain host barriers, such as receptor recognition and cell entry, and can replicate in a range of heterologous cells, tissues, or animal models. However, host switching and stable host adaptation require viruses to cross multiple barriers, including receptor recognition, cell entry, intracellular replication, host antiviral responses, tissue tropism, and individual-to-individual transmission [61]; broad receptor utilization or replication in animal models is not sufficient to demonstrate that a virus can be maintained long-term or establish stable transmission in a new host population [62]. To determine whether specific genetic changes enhance adaptation to a particular host, experiments comparing viruses with and without these changes are needed to assess their ability to enter cells, replicate, or transmit in that host. Direct mechanistic studies, however, remain heavily concentrated on coronaviruses, whereas the determinants of host range for other viruses remain largely unvalidated, constituting an important gap in our understanding of the host adaptation of pangolin-associated viruses.

## 4. Zoonotic Spillover Potential and Contributing Factors

Whether pangolin-associated viruses pose a zoonotic risk in practice depends on whether their biological capacity to infect humans and actual opportunities for human exposure coexist [63]. On the one hand, the virus needs to possess the prerequisites for overcoming human host barriers, including the utilization of human receptors and the capacity for replication in human-derived cells or organoids [56,64]. On the other hand, even if a virus possesses these features, its ability to cause human infection under natural conditions still depends on sufficient contact and exposure opportunities between the animal host and human populations [61,63]. For pangolins, processes such as illegal hunting and trade, long-distance transport, intensive confinement, and rescue or captive care may increase their contact with humans and other animals and alter patterns of pathogen exposure at the human–animal interface [65]. Therefore, assessing the zoonotic spillover potential of pangolin-associated viruses requires consideration not only of biological evidence concerning viral capacity for human infection and the role of the pangolin host, but also of the ecological realities and anthropogenic activities that shape exposure conditions.

### 4.1. Evidence for the Zoonotic Potential of Pangolin-Associated Viruses

Beyond the host range and infectivity of the viruses themselves, the biological characteristics of pangolins as hosts may also influence the infection and maintenance of associated viruses, thereby further affecting their potential zoonotic significance. Pangolins possess an innate immune genetic background that differs from that of most mammals, which may affect their susceptibility to certain viral infections. Genomic studies have revealed that the interferon epsilon gene (*IFNE*) in pangolins has undergone pseudogenization [66]; the *IFIH1* gene, which encodes melanoma differentiation-associated protein 5 (MDA5), and the *ZBP1* gene, which encodes Z-DNA-binding protein 1, have also undergone inactivation or loss during evolution [67]. These changes suggest that pangolins possess unique characteristics in viral nucleic acid sensing and inflammatory regulation, which may result in functional deficiencies in certain innate antiviral sensing and interferon response pathways, thereby creating a relatively permissive host immune environment for certain viruses [18,68]. Furthermore, records of the detection and infection of pangolins with viruses known to infect humans constitute a direct line of evidence for assessing their relevance to human health. In smuggled, rescued, and captive pangolins, viruses known to be capable of infecting humans, such as HRSV, HPIV3, HPIV2, JEV, and CHIKV, have been detected [30,31,32,36]. Among these, the genomes of pangolin-derived HRSV-A, HRSV-B, and HPIV3 share more than 99% nucleotide identity with circulating human strains, and some positive individuals also exhibit high viral sequence abundance or respiratory tract lesions [30,31]. Both JEV and CHIKV are known zoonotic arboviruses. Among them, the pangolin-derived CHIKV strain CHIKV-China/P2020-1 has been successfully isolated as a live virus and can be serially passaged in BHK-21 and Vero cells, confirming its ability to replicate in mammalian cells of different origins [36]. However, the high genetic relatedness of these viruses to human strains does not by itself determine the direction of transmission, and some records may also reflect human-to-pangolin transmission occurring during trade, captive care, or rescue processes [31,69]. In addition to the infection records of the aforementioned viruses known to infect humans, functional evidence directly relevant to human susceptibility has also been obtained for some pangolin-derived viruses. As described in Section 3.2, functional studies of pangolin-derived sarbecoviruses and HKU4-related merbecoviruses have demonstrated human receptor use, replication in human-derived cells or organoids, and infection in transgenic animals expressing human receptors [19,28,58,60]. These findings indicate that pangolins can be infected with or carry multiple groups of viruses of concern to human health and provide multilayered virological and functional evidence for assessing their potential zoonotic risk.

In summary, the zoonotic potential of pangolin-associated viruses has been supported by evidence at multiple levels, ranging from viral detection, natural infection, and live virus isolation to human receptor utilization, replication in human-derived cells or organoids, and infection in animal models. Although the current evidence is insufficient to demonstrate that pangolin-derived viruses can infect humans under natural conditions and establish subsequent transmission, the observed capacities for host infection, human receptor compatibility, and replication in human-derived models underscore the need for continued surveillance of their prevalence, host range, and transmissibility, as well as further assessment of their potential risk of human infection. Representative pangolin-associated viruses with known or potential relevance to human health, together with the supporting evidence categories, are summarized in Table S3.

### 4.2. Ecological and Anthropogenic Drivers of Viral Transmission

Whether the cross-species transmission potential of a virus is realized in practice depends not only on its receptor use and replicative capacity but also on external factors such as host contact frequency, animal health status, and transmission routes [63]. For pangolin-associated viruses, ecological changes, illegal trade, and subsequent rescue and captive care may alter host contact patterns and create opportunities for viral exposure. Ecological changes may first reshape the contact networks between pangolins and other hosts at the source. For instance, deforestation, road and settlement construction, and agricultural expansion can alter the habitat configuration of pangolins and affect their burrow occupancy and spatial distribution [70], causing some pangolin home ranges to extend to the edges of forests, farmlands, plantations, and other human-modified landscapes [71,72]. Long-term telemetry has directly documented that Sunda pangolins move within a forest–oil palm plantation mosaic, with some individuals entering oil palm plantations [73]. Such spatial overlap with human-modified landscapes may increase the opportunities for contact among humans, domestic animals, and wildlife, as well as for pathogen exposure [63,74], and may lead to the relative enrichment of potential zoonotic virus hosts in human-modified landscapes, thereby potentially increasing the risk of zoonotic pathogen transmission [75].

Once pangolins enter illegal trade networks, transmission opportunities may become concentrated. Long-distance, cross-regional, and cross-border transport, often in bags or crates, is associated with dehydration, low body weight, and weakness [27,76]. Studies in other mammalian species have shown that the resulting chronic stress and decline in health status may disrupt host immune function [77,78]. In the context of infection, weakened immune control and nutritional stress may further elevate the levels of viral replication and shedding [79,80]. Meanwhile, pangolins from different geographical origins and trade batches may be housed together in markets, warehouses, or temporary holding facilities [45]. Such a multi-host, high-density, and high-mobility contact environment may not only concentrate viruses originally dispersed across different regions and hosts into the same trade network, but also increase the opportunities for viral spread among animals and for intra- and inter-species transmission [12,81]. At the same time, hunters, transporters, traders, and keepers are more frequently exposed to infected animals and their body fluids, secretions, excretions, and contaminated surfaces, thereby increasing opportunities for human exposure to viruses [65,82]. After confiscated individuals enter rescue or captive facilities, the associated risks do not necessarily cease with the termination of trade activities. Pangolins in different health conditions undergo diagnostic and therapeutic procedures as well as human care during the rescue process [83]. If quarantine, isolation, and environmental disinfection measures are inadequate, opportunities for pathogen transmission among susceptible individuals may increase further, as may the exposure risks for staff and other housed animals [84,85]. The findings from existing coronavirus surveys are consistent with the hypothesis that trade and rescue activities may increase viral exposure. Pangolin sarbecoviruses have been detected multiple times in downstream trade or rescue populations in China and Vietnam [14,15,24]; in contrast, no coronaviruses were detected when screening 334 Sunda pangolins originating from relatively upstream trade links in Malaysia [27]. Although the studies differ in geographic origins, sample sizes, and detection methods, this distribution pattern cannot directly demonstrate viral transmission or accumulation along the trade chain [45]; nevertheless, the available studies suggest that long-distance transport, the convergence of animals from multiple origins, and intensive confinement may increase opportunities for pangolins to be exposed to novel viruses and for infections to spread [27,45,81]. The proposed mechanisms linking stress and impaired immune control to increased viral replication or shedding, and crowding and rescue conditions to viral exposure and transmission, draw partly on evidence from other species. Their application to pangolins should therefore be regarded as biologically plausible hypotheses rather than demonstrated effects in this host.

**Figure 2:**
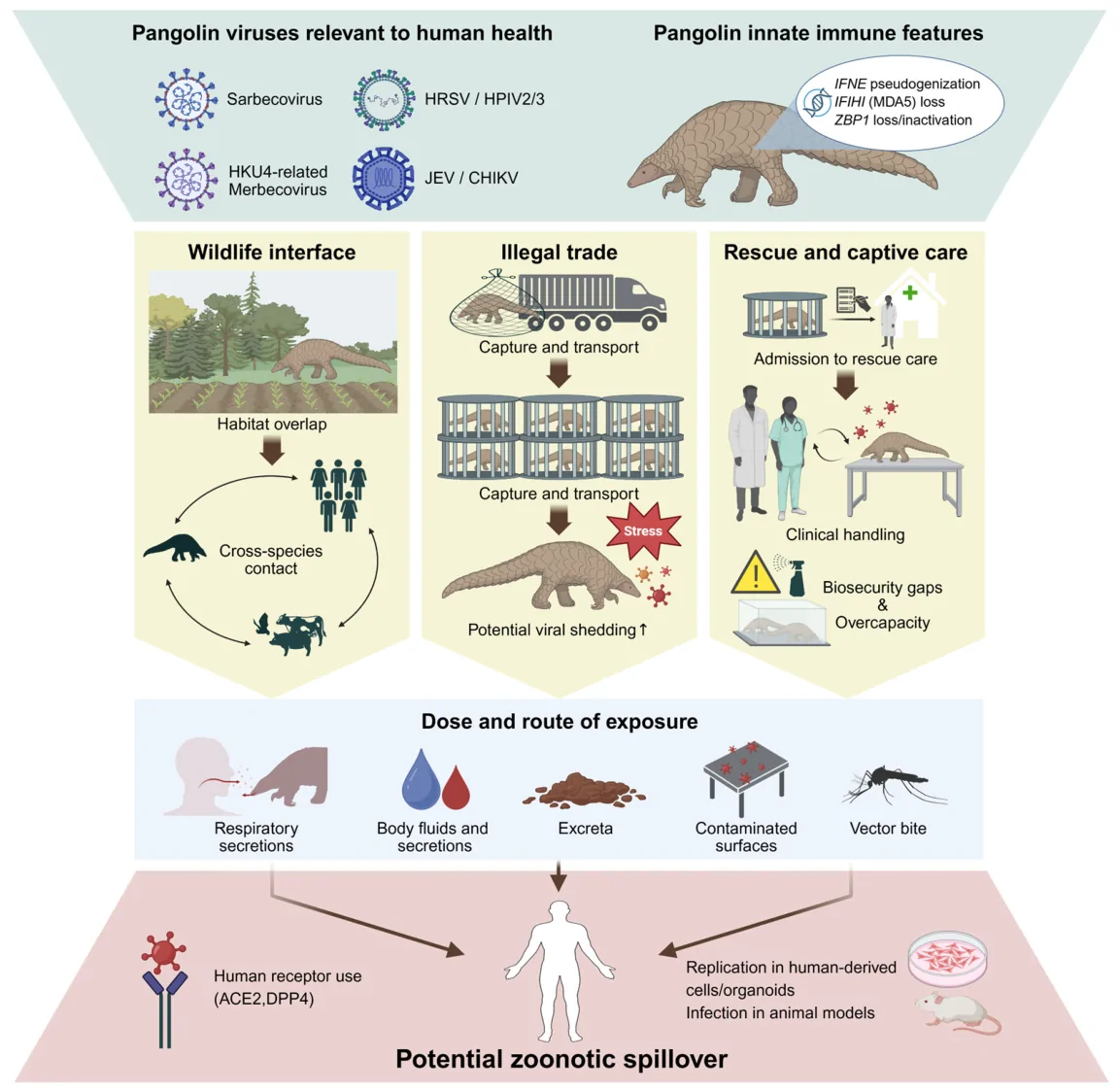
Hypothetical framework for the potential zoonotic spillover of pangolin-associated viruses. Viral and host features are linked to exposure pathways across wildlife interfaces, illegal trade, and rescue or captive care, followed by potential routes of human exposure and evidence relevant to cross-species infection. Arrows indicate hypothesized links and potential exposure or transmission pathways; they do not denote confirmed causal relationships or demonstrated routes of natural transmission from pangolins to humans. Created with BioRender.com.

Overall, ecological disturbance may expand the contact interface between pangolins and humans, domestic animals, and other wildlife at the source, thereby increasing the opportunities for pangolins to encounter heterologous viruses and for cross-species transmission to occur. Illegal trade, centralized rescue, and captive care, in turn, may further concentrate and amplify the associated risks downstream. Therefore, it is necessary to conduct surveillance at the source in pangolin habitats and to treat downstream trade and rescue facilities as critical nodes for screening pangolin-associated viruses, tracking transmission, and assessing potential zoonotic risks. The biological and exposure-related pathways that may contribute to potential zoonotic spillover are summarized in Figure 2.

## 5. Conclusions and Perspectives

Pangolin-associated viruses encompass a wide range of viral taxa and exhibit substantial genetic diversity. Sequence variation and evidence of recombination or reassortment have been reported in selected pangolin-associated viral groups. Some viruses have demonstrated multi-host receptor compatibility and the capacity for replication in heterologous cells or animal models. Moreover, multiple viruses known to infect humans have been detected or recorded in pangolins, and some pangolin-derived viruses have also exhibited human receptor utilization and the ability to infect human-derived models, collectively suggesting that their potential implications for human health warrant sustained attention. However, current research remains heavily concentrated on Sunda and Chinese pangolins, with samples predominantly derived from smuggled, rescued, or captive individuals; studies on other pangolin species and wild populations of known provenance remain extremely limited. At the same time, most viral records remain at the level of nucleic acid sequences, lacking validation through virus isolation, tissue localization, serology, and experimental infection. Unclear geographic origins, unknown exposure histories, and co-infections also constrain the interpretation of natural host associations, transmission directions, and disease causality. Therefore, the diversity of pangolin viruses and the viral groups that truly possess cross-species transmission capacity still require systematic evaluation. As noted by the United Nations Office on Drugs and Crime in its discussion of the illegal pangolin trade and human health risks, pangolins in their natural habitats do not pose a direct threat to humans. Rather, it is their capture, trafficking, and co-trade with other wildlife species that increase the risk of transmission of viruses and other pathogens [86]. In the future, habitat protection, control of illegal trade, viral surveillance in both wild and rescue settings, and functional studies of cross-species transmission should be integrated to achieve the complementary goals of endangered species conservation and public health protection.

## Data Availability

No new data were created or analyzed in this study. Data sharing is not applicable to this article.

## Supplementary Materials

The following supporting information can be downloaded at: https://www.mdpi.com/article/10.3390/ani16193077/s1, Table S1: Search strategy and source documentation for the literature review; Table S2: Host association evidence for the 30 pangolin-associated virus taxa (genera, subgenera, or unresolved lineages) shown in Figure 1; Table S3: Representative pangolin-associated viruses with known or potential relevance to human health.
